# Pathological Role of Interleukin-6 in Psoriatic Arthritis

**DOI:** 10.1155/2012/713618

**Published:** 2012-10-11

**Authors:** Atsushi Ogata, Atsushi Kumanogoh, Toshio Tanaka

**Affiliations:** ^1^Department of Respiratory Medicine, Allergy and Rheumatic Diseases, Osaka University Graduate School of Medicine, Osaka 565-0871, Japan; ^2^Department of Immunopathology, WPI Immunology Frontier Research Center, Osaka University, Osaka 565-0871, Japan; ^3^Department of Clinical Application of Biologics, Osaka University Graduate School of Medicine, Osaka 565-0871, Japan

## Abstract

Psoriatic arthritis (PsA) is a clinical manifestation of psoriatic disease. Although the pathogenesis of PsA remains unknown, PsA can be managed by treatments similar to those used for rheumatoid arthritis (RA). Because interleukin-(IL-) 6 has been suggested to have a pathogenic role in PsA, a humanized anti-IL-6 receptor antibody tocilizumab treatment for PsA was recently tried. However, the efficacy of tocilizumab for PsA was not favorable. This suggests that the pathogenic roles of IL-6 in PsA and RA are different. In RA, tumor necrosis factor (TNF) primarily contributes to the arthritis effector phase and IL-6 contributes to the arthritis priming phase. In PsA, the TNF-related effector phase is similar to that in RA, but the IL-6-related priming phase might not be critical. This paper discusses the role of IL-6 in PsA.

## 1. Introduction

Psoriatic arthritis (PsA) was originally designated as inflammatory arthritis associated with psoriasis that was usually negative for the rheumatoid factor and is now considered as a clinical manifestation of psoriatic disease [1]. Although there are no diagnostic tests for PsA, it is a condition that is distinguishable from rheumatoid arthritis (RA); the characteristic features of PsA and RA are slightly different. In PsA, peripheral arthritis evolves with a distinct joint pattern that possibly involves the distal interphalangeal joints. Dactylitis with enthesitis, involving the entire digit, is a characteristic feature of PsA. Furthermore, articular damage assessed by radiographic erosion is more common in RA and typically reveals an asymmetric pattern in PsA. Despite these differences, the therapeutic options, including tumor necrosis factor (TNF) inhibitors, and the methods for assessing the disease activity are mostly the same. 

Increased production of interleukin-(IL-) 6 is well known in psoriasis and PsA [2, 3]. Mice with epidermal overexpression of IL-6 (K14-IL-6 transgenic mice) exhibit a psoriasis phenotype [4]. The transcription factor signal transducer and activator of transcription 3 (STAT3) is upregulated in psoriasis. IL-6, which induces STAT3 phosphorylation, is also thought to be a potential therapeutic target [5]. In addition, serum IL-6 levels correlate with PsA disease severity [6]. IL-6 is thought to have similar roles in inflammatory arthritis associated with both RA and PsA. This supports the notion that targeted treatments against IL-6 might be effective [7].

## 2. Tocilizumab Treatment for Seronegative Spondyloarthritis

A humanized anti-IL-6 receptor antibody, tocilizumab (TCZ), was recently approved for treating RA patients, and its efficacy for these patients has been demonstrated [8]. The clinical applications of TCZ for PsA have not been well described, although there are some reports on the efficacy of TCZ for seronegative spondyloarthritis (SNSA). SNSA is characterized by the absence of the rheumatoid factor and includes diseases such as PsA. Several case reports have shown favorable outcomes with TCZ treatment for reactive arthritis [9] and ankylosing spondylitis (AS) [10–14].

However, a recent larger case series reported that there were unfavorable outcomes with TCZ treatment for AS. Dudler and Aubry-Roziier reported on the efficacy of TCZ for patients with axial spondyloarthropathies [15]. Among 18 cases, three patients had skin psoriasis. No significant clinical benefits were observed with TCZ for peripheral arthropathies. Del Castillo Piñol et al. reported on five refractory spondyloarthritis (SpA) patients treated with TCZ [16]; a response to TCZ was found in only one of the five severe cases of axial SpA. Lekpa et al. reported on 21 spondyloarthritis patients who were treated with TCZ, for whom anti-TNF-*α* therapy had failed [17]. Although TCZ decreased acute-phase reactions, TCZ failed to substantially improve axial spondyloarthritis and was inconsistently effective for peripheral spondyloarthritis. 

More recently, the results of two randomized control trials (RCTs) that used IL-6 inhibitors were reported. Sieper et al. reported on a phase 2 study of TCZ for AS [18]. They enrolled 102 AS patients, and 51 patients were treated with TCZ for 12 weeks. Although the C-reactive protein (CRP) levels declined, AS symptoms were not improved. The efficacy of TCZ for treating AS was not demonstrated in this RCT. In addition, a phase 2 RCT of another IL-6 receptor antibody, sarilumab, also failed to demonstrate its efficacy in AS patients assessed by their 20% improvement in Assessment of Ankylosing Spondylitis (ASAS20) responses at 12 weeks [19]. 

## 3. TCZ Treatment for Psoriatic Arthritis

**Figure 1:**
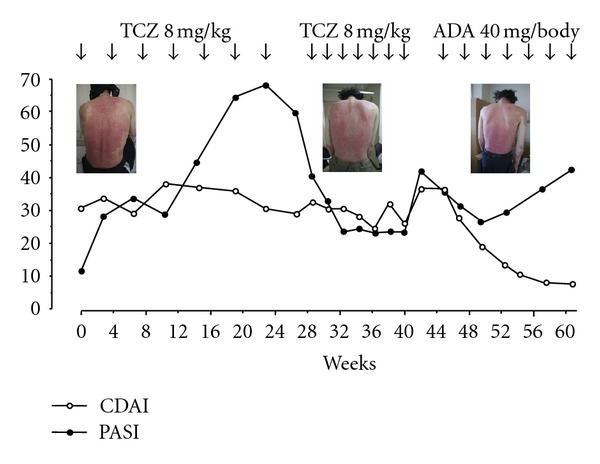
Changes in the clinical disease activity index (CDAI) and the psoriasis area-and-severity index (PASI) score for case 1 during tocilizumab and adalimumab therapy.

We recently reported on two PsA patients who were treated with TCZ [20]. The first was a 35-year-old man. He was started on 8 mg/kg every 4 weeks. His clinical course is shown in Figure 1. Before TCZ treatment, his clinical disease activity index (CDAI) was 30.8, and his Psoriasis Area and Severity Index (PASI) was 11.3. After seven TCZ infusions, his CRP levels had not improved (7.20–5.71 mg/dL), suggesting that a 4-week interval between the TCZ infusions was not sufficient to inhibit the IL-6 activity in this patient. After a 2-week interval between infusions, his CRP levels returned to normal. However, both his CDAI and PASI had not improved. Adalimumab was then initiated. Although his CRP levels increased (1.31 mg/dL) and his PASI did not improve rapidly, his CDAI was significantly improved.

**Figure 2:**
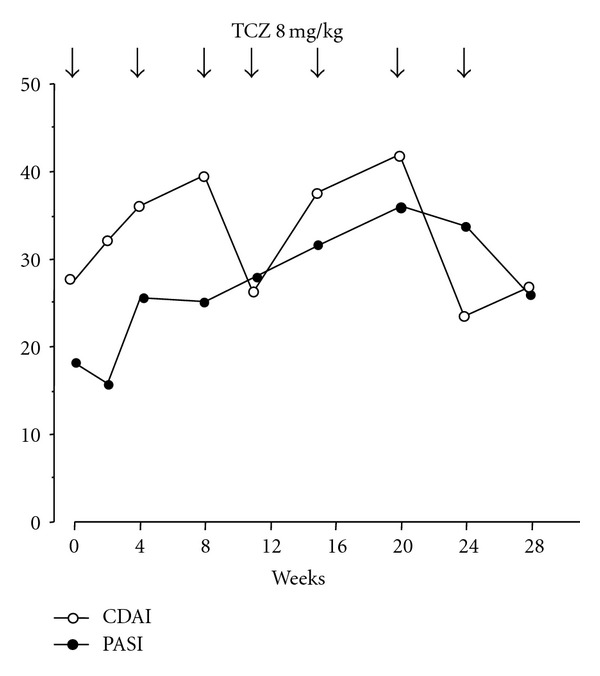
Changes in the clinical disease activity index (CDAI) and the psoriasis area-and-severity index (PASI) score for case 2 during tocilizumab therapy.

The second case was a 28-year-old man. TCZ was started at 8 mg/kg every 4 weeks. His clinical course is shown in Figure 2. His CRP levels normalized; however, his clinical symptoms as assessed by CDAI and PASI remained unimproved after seven TCZ infusions. In both cases, TCZ treatment resulted in normalized CRP levels, which suggested that TCZ had completely inhibited IL-6 signaling. However, the clinical symptoms of PsA had not improved. TNF inhibitors were efficacious in both cases. These results suggest that TCZ may not be a new therapeutic option for PsA. However, the patient with a history of HLA-B27 positive AS and Crohn's disease from Brulhart et al.'s paper also had psoriasis [10]. While the efficacy of tocilizumab infused at 8 mg/kg monthly was not observed, the infusion frequency to every 15 days induced rapid biological and progressive clinical improvement. It was reported that after 11 months, the patient remained well with no tender and swollen joints with normalization of inflammatory markers and psoriasis skin lesions have completely resolved [10]. In contrast, there are some recent descriptions of patients with RA or adult-onset Still's disease that developed psoriatic skin lesions following treatment with tocilizumab [21, 22]. However, these were only representative cases and randomized controlled trials are needed. 

## 4. Adaptive Immune Responses in PsA

The pathogenic role of the adaptive immune system in PsA is different from that in RA. It is well known that CD4+ T cells are important in RA. In contrast, there is increasing evidence that PsA is an autoimmune disease in which CD8+ T cells play a key role [23]. Indeed, the de novo appearance of PsA in patients with acquired immunodeficiency syndrome and advanced depletion of CD4+ T cells during the early stages of the human immunodeficiency virus pandemic suggested different roles of T cells in PsA than those in RA and systemic lupus erythematosus (SLE), which are both ameliorated by a loss of CD4+ T cells [24]. A TNF-overexpressing mouse model (TnfΔARE mice) is a model of spondyloarthritic disease that is uniquely associated with Crohn's-like inflammatory bowel disease [25]. This mouse model develops chronic polyarthritis beginning at 5-6 weeks of age and intestinal disease beginning at week 6. A requirement for CD8+ effector functions has been reported for this model [26]. These findings indicate the importance of CD8 T cells in SNSA.

Favorable outcomes with abatacept for PsA were recently reported [27]. Abatacept (CTLA-4-Ig) is a biological agent constructed by genetically fusing the external domain of human CTLA-4 and a fragment of the Fc domain of human immunoglobulin (Ig) G1. As with native CTLA-4, abatacept first binds to CD80/CD86 on antigen-presenting cells and then to CD28 on T cells. Interfering with the CD28 costimulation pathway results in inhibiting antigen-dependent T-cell activation [28]. Abatacept treatment inhibits the T- and B-cell activity in the synovial fluid of RA patients by downregulating the expressions of both CD4 and CD8 [27]. Because CTLA-4-Ig inhibits CD8+ T cells' cytotoxic responses [29], abatacept may improve PsA symptoms by inhibiting CD8+ T cell-activation.

In a psoriasis animal model, skin lesions spontaneously developed when symptomless prepsoriatic human skin was engrafted onto AGR129 mice that were deficient in type I and type II interferon receptors and the recombination activating gene 2 [30]. In this model, resident human T cells in prepsoriatic skin underwent local proliferation in the engraftment. Although CD4+ cells were predominantly found in the dermis, CD8+ cells were also located predominantly in the epidermis or the dermoepidermal junction zone. Long-lived, sessile, and resident T cells may be important in the pathogenesis of psoriasis. In addition, human normal skin contains significant numbers of resident T cells, including Th17 cells [31]. 

Acquired immunity, especially that mediated by the Th17/IL-23 axis, plays an important role in the inflammatory pathology observed in psoriasis and PsA [32]. Th17 cells are a separate lineage of T cells that depend upon IL-23 for their development, survival, and proliferation [33]. Increase in the number of Th17 cells is found in psoriatic lesions [34]. Th17 cells produce the cytokines IL-17, TNF-*α*, IL-21, and IL-22. IL-22 induces human keratinocyte proliferation [35]. IL-17 is a proinflammatory cytokine that promotes migration of neutrophils into the psoriatic lesions. Injecting IL-23 into the skin of mice induced dermal changes that are seen in psoriasis, and these effects were mediated by IL-22 [36, 37]. In the skin, dendritic cells and keratinocytes produce increased amounts of IL-23, a cytokine that supports the development and proliferation of Th17 cells. IL-6 is also involved in autoimmunity by altering the balance of Th17 cells by inducing the differentiation of Th17 cells from naïve CD4+ T cells [38]. Although IL-6 is a possible inducer of Th17 cells from naïve T cells, IL-23 may contribute to the activation of skin-resident Th17 cells that are already differentiated.

Indeed, a recent genome wide association study (GWAS) identified a signaling network of adaptive immune responses in psoriasis that involved CD8+ T cells and Th17 cells [39]. In PsA, T-cell activation and inflammatory cytokine production might occur in the dermis with subsequent migration to the joint, or CD8+ cytotoxic T cells might originate in the synovium. In contrast to RA, contribution of the IL-6 priming phase of arthritis may not be important in PsA.

## 5. Innate Immune Responses in PsA

The previously cited GWAS also identified skin barrier functions and innate immunity responses, involving nuclear factor-kappa B (NF*κ*B) and interferon signaling in PsA [39]. Innate immunity is the first line of defense against invading organisms, which involves activating intracellular regulators such as NF*κ*B. The expressions of numerous target genes involved in the pathogenesis of inflammatory diseases, including TNF, IL-1, and IL-17, are triggered by NF*κ*B. Several factors may exacerbate these manifestations or even trigger the disease, such as traumatic injury to the skin, physical and psychological stress, cold weather, excessive alcohol consumption, and drugs like lithium and beta blockers [40, 41]. 

The role of physical injury, including trauma, in PsA has also been the subject of some interest. It is intriguing to speculate that the Koebner phenomenon, a recognized feature of skin psoriasis, may also occur in peripheral joints [42]. With regard to the pathogenesis of trauma-induced PsA, the “deep Koebner's phenomenon” has been proposed [43]. Microbial infections have also been known to trigger psoriasis and PsA [44]. In addition, IFN-*α* is a well-known trigger of PsA [45]. These findings suggest that innate immunity is also involved in PsA. Inflammation derived from the direct activation of innate immunity may result in the production of TNF and the development of arthritis. 

## 6. Pathogenic Role of IL-6 in PsA

The precise mechanisms by which IL-6 blockade leads to improvements in RA are not well understood [46]. IL-6 promotes synovitis by inducing neovascularization, infiltration of inflammatory cells, and synovial hyperplasia. IL-6 also causes bone resorption by inducing osteoclast formation via the induction of the receptor activator of NF*κ*B ligand (RANKL) in synovial cells and cartilage degeneration by inducing the production of matrix metalloproteinases in synovial cells and chondrocytes. Moreover, IL-6 is involved in autoimmunity by altering the balance of Th17 cells by inducing the differentiation of Th17 cells from naïve CD4+ T cells. IL-6 blockade inhibits type II collagen-induced arthritis and requires CD4 T cells, which leads to the production of anti-type II collagen IgG [47]. However, another arthritis model, anti-type II collagen antibody-induced arthritis, bypasses the priming phase of T-cell-dependent antibody generation and is not suppressed in IL-6−/− mice [48]. These findings indicate that IL-6 is involved in the priming phase of RA but not in the effector phase of RA. Therefore, the major mechanism of TCZ is inhibiting the immune activation that leads to the development of RA [49].

IL-1R antagonist (−/−) mice spontaneously develop autoimmune diseases such as arthritis and dermatitis that histologically resemble human psoriasis [50]. In this model, the deficiency of TNF, but not IL-6, suppressed the development of arthritis and skin inflammation [51]. This suggests that TNF, but not IL-6, is important in the pathogenesis of PsA. 

A recently proposed model for the pathogenesis of PsA is that the frequent microdamage and tissue repair at normal enthesis attachment sites in healthy joints results in PsA pathogenesis. This model incorporates the concept of autoinflammation in which tissue specific factors, including microtrauma, lead to regional innate immunity activation and persistent inflammation as an alternative to primary immunopathology driven by T- and B-cell abnormalities [52].

**Figure 3:**
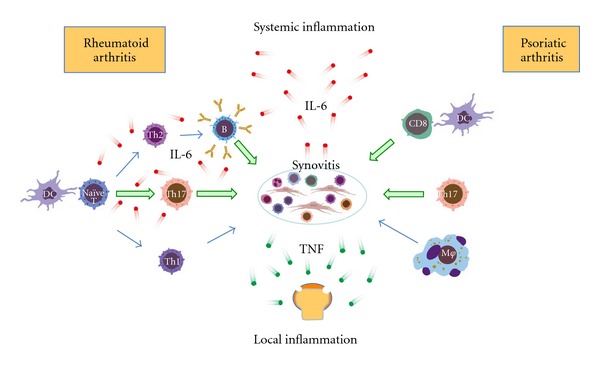
Pathogenic roles of IL-6 in RA and PsA. IL-6 contributes to the priming phase of RA. Because an IL-6-independent innate immune mechanism primarily contributes to PsA, the roles of IL-6 in the pathogenesis of PsA are not critical.

The difference in the pathogenesis of RA and PsA are summarized in Figure 3. In RA, CTLA-4-dependent antigen presentation to CD4+ cells and IL-6-dependent Th17 differentiation induces synovitis. In addition, B cells contribute to the pathogenesis of RA. In PsA, IL-23-dependent differentiated skin-resident Th17 cells, activated CD8+ cytotoxic cells, and directly activated macrophages are mainly involved in synovitis development. Because synovitis that produces TNF contributes to the effector phase of arthritis in both RA and PsA, TNF inhibitors are effective for both types of arthritis. IL-6 primarily contributes to the priming phase of synovitis in RA. Systemic inflammation induced by IL-6 is not the main mechanism of TCZ action in treating RA. Although systemic inflammation is inhibited by TCZ, it does not inhibit the associated arthritis. 

## 7. Conclusions

Although TCZ has shown significant efficacy for treating RA, TCZ treatment has not shown favorable outcomes in PsA. This suggests that the roles of IL-6 in RA and PsA are different. Although the exact reason for ineffectiveness of TCZ in PsA is unclear, IL-6-dependent immune response activation may not be important in PsA.
